# Rehabilitation and release of White-tailed Eagles (*Haliaeetus albicilla*) in Bulgaria: A case study

**DOI:** 10.3897/BDJ.13.e167730

**Published:** 2025-10-31

**Authors:** Rusko Petrov, Vania Marutsova, Dimitar Popov, Volen Arkumarev, Anton Stamenov, Gradimir Gradev, Ivaylo Klisurov

**Affiliations:** 1 Trakia Univeristy, Stara Zagora, Bulgaria Trakia Univeristy Stara Zagora Bulgaria; 2 Green Balkans – Stara Zagora NGO, Stara Zagora, Bulgaria Green Balkans – Stara Zagora NGO Stara Zagora Bulgaria; 3 Bulgarian Society for the Protection of Birds/ BirdLife Bulgaria, Sofia, Bulgaria Bulgarian Society for the Protection of Birds/ BirdLife Bulgaria Sofia Bulgaria; 4 Agricultural University Plovdiv, Plovdiv, Bulgaria Agricultural University Plovdiv Plovdiv Bulgaria

**Keywords:** White-tailed Eagle, *Haliaeetus albicilla*, wildlife rehabilitation, GPS tracking

## Abstract

The White-tailed Eagle (*Haliaeetus albicilla*) is a top predator and may serve as an indicator of environmental health. The species is listed as Least Concern in the IUCN Red List of Threatened Species. As of 2020, the population of White-tailed Eagles in Bulgaria was estimated at 43-60 breeding pairs. Conservation efforts were undertaken in the country, including rescue, rehabilitation and release of birds back into the wild. Soft release was used for the first time in Bulgaria for White-tailed Eagles at two different locations: Karandila in Sinite Kamani Nature Park (Sliven Region) and near Potochnitsa Village (Kardzhali Region). The current study tracks the rehabilitation of four immature eagles tagged with GPS-GSM transmitters, their adaptive capacity and the success of their release back into their natural environment.

## Introduction

### Status

The White-tailed Eagle (*Haliaeetus albicilla*) is a large diurnal bird of prey, belonging to the order Falconiformes and the family Accipitridae. It is found in Europe, Asia, Africa and Greenland. It is listed in the IUCN Red List of Threatened Species as Least Concern (LC) (BirdLife International 2021b). In Bulgaria, its conservation status is Vulnerable (VU) [B2+c(i, iii)], with protection under the EU Birds Directive (Ivanov et al. 2014). The White-tailed Eagle population drastically declined in the late 20^th^ century in the Baltic Sea region due to hunting and pollution with organochlorine pesticides. However, following extensive conservation measures in the 1980s, its population began to recover (Stjernberg et al. 2007, Ekblad et al. 2020). Both the European and Bulgarian populations of the species are currently considered to be increasing (Chesmedjiev and Demerdzhiev 2020, BirdLife International 2021a). The population in Western Europe is recovering due to re-introduction efforts in England, Scotland and Ireland and is also increasing in Finland (O’Rourke 2014, Högmander et al. 2020). In Bulgaria, the White-tailed Eagle is a protected species (Biodiversity Act, Annexes II and III). Its nesting habitats include the Black Sea coast, the Danube islands (including the protected area "Belene Islands Complex"), the Burgas Lakes, Durankulak Lake, the valleys of the rivers Tundzha, Arda, Kamchiya and Ropotamo, as well as around some larger inland reservoirs, such as Rozov Kladenets, Trakiets, Zhrebchevo, Pyasachnik, Studen Kladenets, Tsonevo, Ovcharitsa, Ivaylovgrad and Koprinka, amongst others (Simeonov et al. 1990, Kostadinova 1997, Todorov et al. 2015, Cheshmedzhiev et al. 2019). According to Chesmedjiev and Demerdzhiev (2020), the total number of nesting pairs in Bulgaria is 43-60. In neighbouring countries, the White-tailed Eagle population is stable. In Turkey and Greece, there are 15 and 6 pairs, respectively, in Serbia - 90 pairs of nesting birds and, along the Danube River in Romania - 40 pairs (Birdlife International 2004, Probst and Gaborik 2011, Kiss et al. 2013).

### Threats

**Figure 1.**
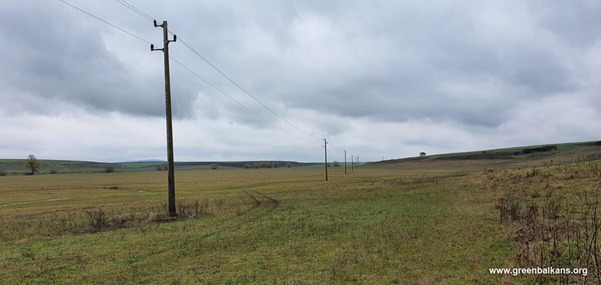
Medium voltage 20 kW electric pylons - the most dangerous type for large birds in Bulgaria.

Amongst the main threats to the White-tailed Eagle populations are habitat loss caused by the draining of wetlands, illegal logging of riparian forests (Probst and Gaborik 2011), illegal poisoning (Stjernberg et al. 2007, Mirmigkou and de Boer 2015, Isomursu et al. 2018), industrialisation (Balotari-Chiebao et al. 2021), poaching, illegal egg collection, hunting with lead ammunition, alongside other factors (Panter et al. 2022, Rozsypalová et al. 2024). A serious threat to the White-tailed Eagle is overhead power lines, where electrocution may occur when eagles perch on the poles (Bevanger et al. 2010, Isomursu et al. 2018; Bernardino et al. 2018, Sarasola et al. 2020, Rozsypalová et al. 2022). In Bulgaria, a high percentage of the overhead power lines are hazardous for large raptors and storks, causing the death of thousands of birds every year (Demerdzhiev et al. 2009) (Fig. 1).

## Material and methods

Between 1995 and 2025, eight White-tailed eagles in total have been admitted to the clinic of the Wildlife Rehabilitation and Breeding Centre of Green Balkans - Stara Zagora NGO. The present study was conducted between 2019 and 2022 in Bulgaria. It includes four White-tailed Eagle chicks, which fell from their nests due to adverse weather conditions. Two of them were siblings from a nest near the village of Benkovski, Stara Zagora Region. The third eagle was a single chick from a nest near the village of Kirilovo, Yambol Region, the fourth - from Tenevo, Yambol. The first two chicks were rehabilitated and soft-released and the other two were hard-released following treatment and rehabilitation. The four birds were equipped with GPS-GSM transmitters prior to their release.

## Results

Description of the four cases of rehabilitation and release of White-tailed Eagles in Bulgaria:

### Case study 1: White-tailed Eagle № 551/2019

On 1 June 2019, as a result of strong winds, the two White-tailed Eagle chicks fell from their nest. They were found and taken to the Wildlife Rehabilitation and Breeding Centre of Green Balkans - Stara Zagora NGO. A clinical examination revealed that the two juvenile eagles were approximately 4-6 days apart in age. The elder sibling was assigned registration number 551/2019. It was admitted with a weight of 4.320 kg, blood in the oral cavity and wheezing sounds while breathing. A radiological examination was performed, which revealed a right-sided pelvic fracture.

After stabilising its condition, on 14 June 2019, surgery was performed – osteosynthesis with cerclage. The surgical treatment was accompanied by medication therapy (anti-inflammatories, pain medications, fluids). During this period, the bird refused to eat on its own and hand-feeding was required. A recovery period followed in the intensive care unit. Once the eagle started to feed on its own, on 15 July 2019, it was moved to a larger aviary. It weighed 3.700 kg.

During a clinical examination on 22 August 2019, it was found that the tail feathers were broken due to stress. To stimulate growth, the tail feathers were plucked one by one. Once the new feathers grew, the remaining broken feathers were plucked sequentially. During a secondary examination on 21 October 2019, broken primary feathers were discovered on both wings – four on each wing. These broken feathers were removed as well. During this period, the bird was using both legs and was feeding independently. The bird was scheduled for release in April 2020.

On 29 April 2020, at nearly one year of age, the eagle was transferred from the Rehabilitation Centre to an adaptation aviary near the Karandila area in the "Sinite kamani" Nature Park, located above the City of Sliven. The bird remained there for three weeks in order to adapt to the area. A vulture feeding site was located near the aviary, providing food to the young eagle in the early days of its release. The eagle was equipped with a satellite transmitter (Ornitela OT-20-3GC, 20 g) to track its movements and monitor its adaptation in the wild.

**Figure 2.**
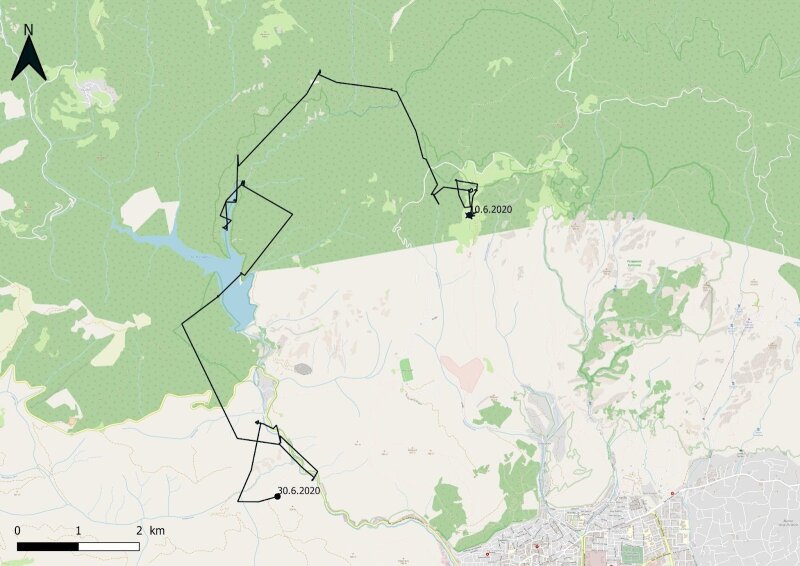
The route of White-tailed Eagle № 551/2019.

The young eagle was released from the aviary in May 2020 (Fig. 2). Using telemetry data, the bird was located two months later in a visibly exhausted state and was re-admitted to the Rehabilitation Centre on 2 July 2020. A clinical examination revealed no fractures, but the eagle was weak and emaciated. It was placed under observation and given a general strengthening therapy. A week after staying in the intensive care unit, the eagle was moved back to a large aviary, now weighing 4.530 kg.

During a prophylactic check-up on 27 October 2020, it was noted that the eagle had lost the primary feathers on its left wing due to moulting. The feather replacement continued on the other wing throughout 2021. By November of the same year, most feathers had grown back, but some remained short.

On 8 November 2022, the bird was transferred to GREFA (Group for the Rehabilitation of Local Fauna and Its Habitats) in Spain. The eagle was transferred to a captive facility and integrated into a captive breeding programme, with the goal of releasing its offspring into the wild.

### Case study 2: White-tailed Eagle № 552/2019

The younger sibling, with registration number 552/2019, was admitted without visible injuries or trauma, weighing 3.550 kg. A radiological examination was conducted, which confirmed that there were no injuries from the fall.

**Figure 3.**
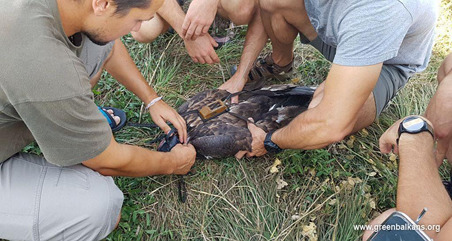
Placing a satellite transmitter on White-tailed Eagle.

**Figure 4.**
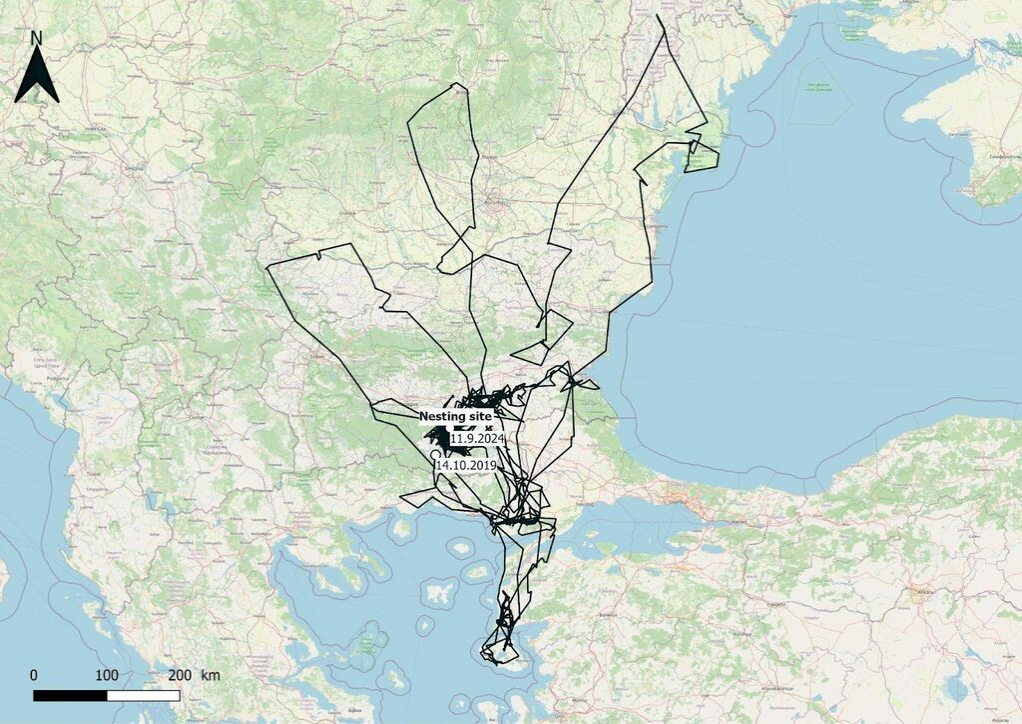
The route of White-tailed Eagle № 552/2019.

The bird was kept for three months until fully grown. After this period, the eagle was transferred from the Green Balkans Wildlife Rehabilitation and Breeding Centre to an adaptation aviary near the village of Potochnitsa (Haskovo Region), in the Eastern Rhodopes, where it stayed for two months. A vulture feeding site is located close to the aviary, providing food for the young eagle during its first days after the release. The eagle was equipped with a satellite transmitter to track its behaviour and movements (Fig. 3). It was released from the adaptation aviary in October 2019. The data from the satellite transmitter (47 g solar-powered GPS/Argos transmitter manufactured by Microwave telemetry) enabled tracking of the movements of eagle № 552/2019 over the next 5 years (Fig. 4). In its first years, the eagle travelled longer distances and visited Ukraine, Moldova, Romania, Greece and Turkey. For most of the summer and winter periods, the eagle spent in Thrace and the Eastern Rhodopes in Bulgaria. In 2022 and 2023, the bird settled near the water reservoir Trakiets near the City of Haskovo, where it was observed together with other immature White-tailed Eagles. In 2023, it was observed with a partner exhibiting territorial behaviour. In January 2024, the nest of the pair was discovered and they raised at least one fledgling.

### Case study 3: White-tailed Eagle № 1773/2020

On 25 November 2020, after а strong storm during the night, a White-tailed Eagle was found to have fallen from its nest. It was hatched by a pair nesting near the village of Kirilovo, Yambol Region. The bird was taken to the Green Balkans Wildlife Rehabilitation and Breeding Centre and was registered with number 1773/2020.

**Figure 5.**
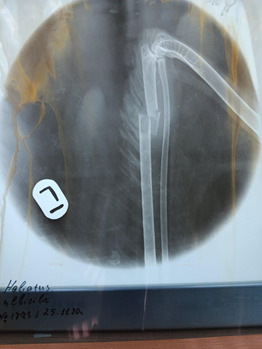
White-tailed Eagle № 1773/2020 with a fragmented fracture of the ulna and radius on the left wing.

After a clinical examination and radiological investigation, it was determined that the bird had an undisplaced fragmented fracture of the left wing, affecting both the radius and the ulna bones (Fig. 5). Following therapy and complete recovery, the eagle was tagged with a transmitter (Ornitela OT-20-3GC, 20 g) and hard-released on 11 February 2021, in the vicinity of the village of Kirilovo, Yambol Region close to its natal site.

**Figure 6.**
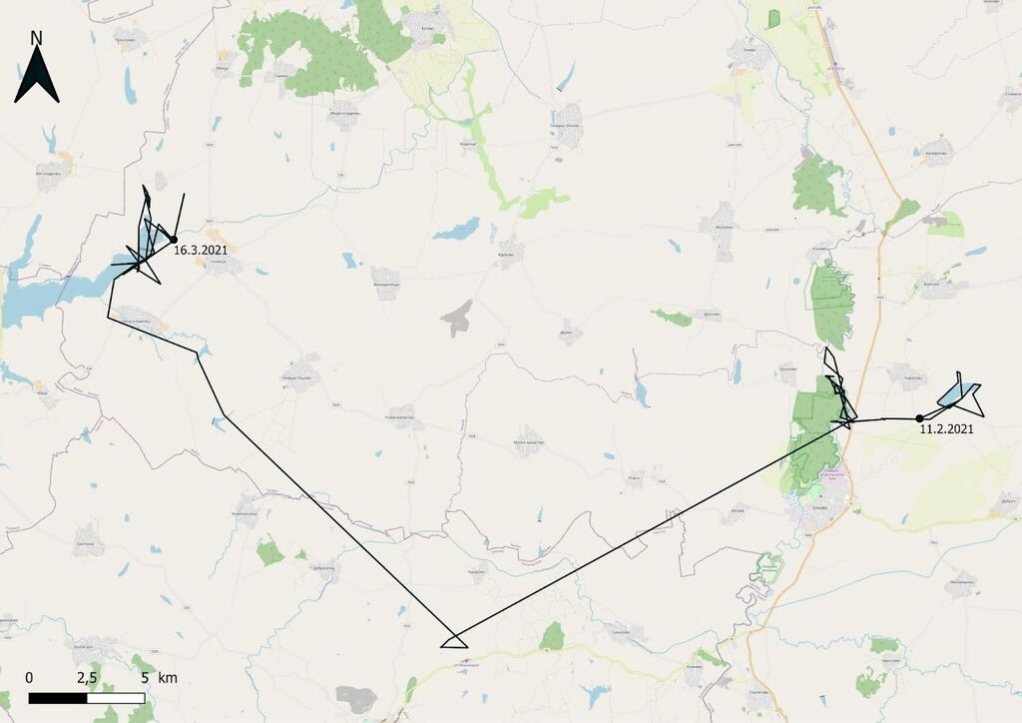
The route of White-tailed Eagle № 1773/2020.

**Figure 7.**
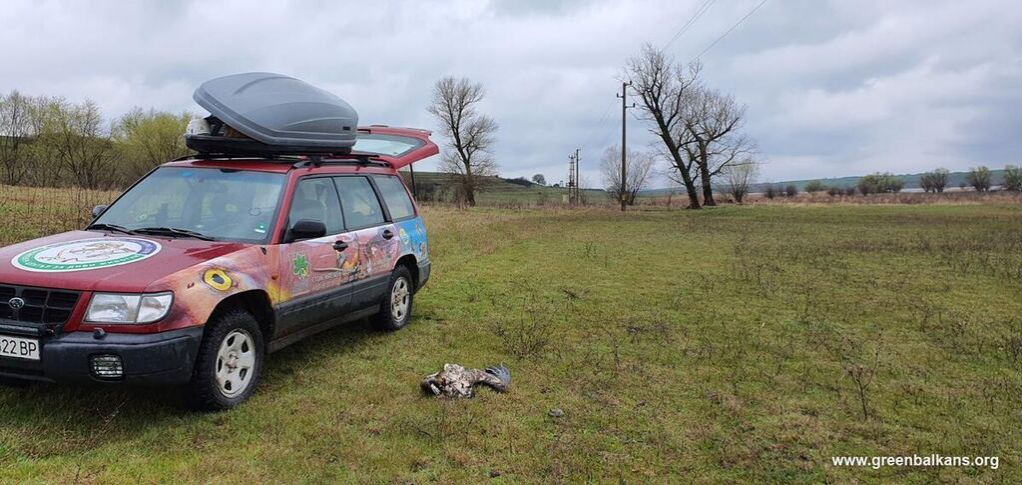
White-tailed Eagle № 1773/2020 found dead near a power line.

The route of the young eagle was tracked over the next month (Fig. 6). It visited Dolna Topchiya Reserve along Tundzha River and moved to Ovcharitsa Reservoir - a site occupied by a breeding pair. In March 2021, the GPS transmitter indicated a lack of movement and a team visited the site. The eagle was found dead with burnt feet under a power line (Fig. 7).

A necropsy confirmed that the cause of death was an electric shock.

### Case study 4: White-tailed Eagle № 1934/2025

**Figure 8.**
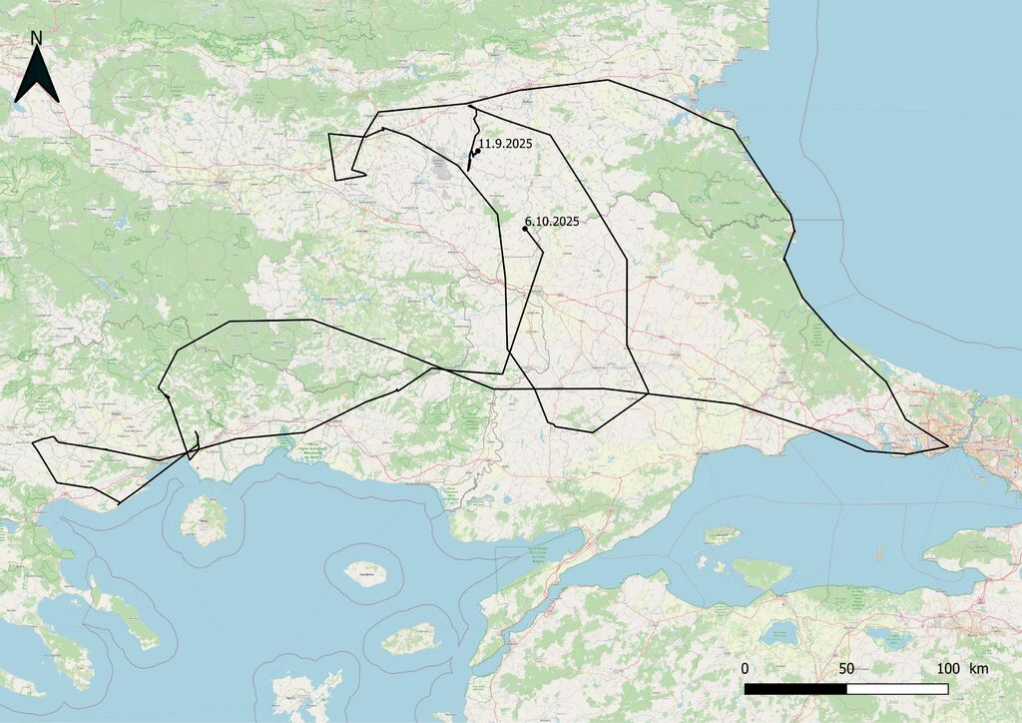
The route of White-tailed Eagle № 1934/2025.

It was found near the village of Tenevo, Yambol Region and admitted to the Green Balkans Wildlife Rehabilitation and Breeding Centre on 23 July 2025. Its right eye had a damaged pupil and it had a fracture of the left shoulder. Released on 11 September 2025, near the Ovcharitsa Dam with an Ornitela transmitter (Ornitela OT-20-3GC, 20 g). Weight at release - 4500 g. The eagle died from electrocution on 6 October 2025 (Fig. 8). It was hard-released.

## Discussion

Around the world, there are many rehabilitation centres for birds of prey that continuously respond to reports of distressed birds and provide care for them. Only 40% of these birds are later returned to their natural habitat (Rozsypalová et al. 2022). The process of rehabilitating wild birds is difficult and often costly (Rozsypalová et al. 2024). Successful recovery, the study of adaptive capabilities and survival after release into the wild are long-term processes that require both time and specialised equipment. GPS tracking enables successful long-distance and long-term monitoring of released birds (Csermely 2000). According to Martell et al. (2000), the evaluation of a successful bird release into the wild is based on three parameters: survival, reproductive success and spatiotemporal activity.

Our study tracked the successful rehabilitation and re-integration processes of four White-tailed Eagles admitted to the Green Balkans Wildlife Rehabilitation and Breeding Centre and soft-released into the wild - performed for the first time for this species in Bulgaria.

Several nature conservation NGOs in Bulgaria are working closely with power distribution companies to secure these poles by using various methods, such as installing protective insulation and mounting special platforms that provide resting spaces for the eagles and allow them to perch at a safe distance from the wires. Efforts are underway to replace overhead power lines with fully insulated ones – the PAS system, which significantly reduces the risk of electrocution. The most effective approach for bird protection is the use of underground power cables, which replace the dangerous overhead power lines. This last method eliminates the risk to birds, but is the most complex and costly to implement. Securing dangerous electricity poles is one of the measures with a favourable conservation effect on the White-tailed Eagle, as it reduces bird mortality from electrocution.

Human activity has always played a key role in causing injuries and mortality in White-tailed Eagles in their natural habitats (Komosa et al. 2018, Rozsypalová et al. 2022, Probst et al. 2024). Annual mortality due to these factors ranges between 17% and 74% (Millsap et al. 2022, Rozsypalová et al. 2024). Our research data confirm that eagles rehabilitated in rescue centres and soft-released can survive and reproduce successfully in the wild. This is a conclusion also supported by other authors (Csermely 2000, Rozsypalová et al. 2024). The reproductive success rate for White-tailed Eagles released from rescue centres is 70% in Austria (Probst and Pichler 2017), 94% in Slovakia (Chavko 2024) and 64% according to Rozsypalová et al. (2024).

## Conclusions

The results of the study, conducted for the first time in Bulgaria, show that the application of the soft release method of two rehabilitated White-tailed Eagles may lead to successful survival and reproduction of the released individuals in the wild. A more in-depth study with larger sample size is needed to confirm the success of this methodology. The observed behaviour and successful adaptation of the eagles after their release (following severe fractures of wings and pelvic bones) confirm the effectiveness of these methods and reinforce the role of wildlife rescue centres in species conservation. Only through proper care, technology and collaboration can rehabilitated White-tailed Eagles successfully return to the wild and even reproduce, thereby contributing to species conservation at both national and international levels. However, the human factor remains a significant threat to the population of this top predator, underscoring the need for targeted efforts to protect their natural habitats and mitigate anthropogenic risks, such as unsafe power lines and poaching.
